# Mendelian randomization identifies circulating miRNAs as causal mediators of gastric cancer susceptibility and survival outcomes

**DOI:** 10.1097/MD.0000000000046833

**Published:** 2026-01-02

**Authors:** Yejue Lin, Ming Luo

**Affiliations:** a Department of Thyroid and Breast Surgery, First Affiliated Hospital of Huzhou University, Huzhou, Zhejiang, China.

**Keywords:** expression quantitative trait loci, gastric cancer, Mendelian randomization, miRNA, prognostic relevance

## Abstract

Gastric cancer (GC) remains a leading cause of cancer mortality, yet the causal roles of microRNAs (miRNAs) in its pathogenesis are poorly characterized. While observational studies implicate miRNAs in GC progression, confounding biases and tissue-specific limitations hinder causal inference and clinical translation. We conducted a 2-sample Mendelian randomization (MR) analysis using genetic instruments derived from plasma miRNA expression quantitative trait loci (eQTLs). Summary-level data for miRNA-eQTLs were obtained from a study by Huan et al. (involving 5239 individuals and 280 miRNAs), while genetic associations with GC were sourced from 3 independent genome-wide association studies (ebi-a-GCST90018849, ebi-a-GCST90018629, and bbj-a-119) accessed via the IEU OpenGWAS Project. Instrumental variables were constructed using miRNA-eQTLs that reached significance at a false discovery rate (FDR <  0.1. Causal estimates were primarily generated using inverse-variance weighted regression, supplemented by MR-Egger regression to assess and adjust for potential pleiotropy. Sensitivity analyses, including leave-one-out validation, were performed to evaluate the robustness of the findings. Experimentally validated targets were analyzed for differential expression, prognostic relevance, and somatic mutations. Functional enrichment and pan-cancer analyses were conducted to delineate oncogenic mechanisms. MR analysis revealed 5 plasma miRNAs with consistent causal effects on GC risk: hsa-miR-127-3p, hsa-miR-370-3p, hsa-miR-382-5p, hsa-miR-409-3p, and hsa-miR-654-5p. All 5 miRNAs conferred increased risk (ORs 1.021–1.037, all  ≤0.0025) across the 3 cohorts (ebi-a-GCST90018849, ebi-a-GCST90018629, bbj-a-119). These miRNAs collectively targeted 549 genes, of which 76 were differentially expressed in GC tissues. Seventeen dysregulated targets showed prognostic significance, with enrichment in immune regulation (T/B cell receptor signaling) and cancer pathways. In GC, miR-409-3p overexpression independently predicted poor survival (H =  1.55, *P* = .0098) and inversely correlated with multiple targets (XKR4, F2, ATAD5, GNAL, GDNF, UNC13A, and ELL2). Pan-cancer analysis revealed oncogenic roles for causal miRNAs in 16 malignancies, with miR-409-3p showing GC-specific prognostic significance. This MR study establishes plasma miRNAs as causal mediators of gastric carcinogenesis, with miR-409-3p emerging as a key prognostic biomarker. The identified miRNA-target networks highlight actionable pathways for therapeutic intervention, bridging genetic epidemiology with functional genomics in GC precision oncology.

## 1. Introduction

Gastric cancer (GC) remains a leading cause of global cancer-related mortality, with over 1 million new cases and 769,000 deaths annually, highlighting its aggressive biology and limited therapeutic advancements.^[1]^ Despite progress in diagnostic and therapeutic strategies, the molecular mechanisms driving GC pathogenesis, metastasis, and chemoresistance remain incompletely understood. Recent studies underscore the pivotal role of microRNAs (miRNAs) in GC biology, where they regulate gene expression at posttranscriptional levels. For instance, miRNA-145-5p suppresses GC progression by targeting SERPINE1 to inhibit the ERK1/2 pathway,^[2]^ while miR-194-2HG modulates miRNA biogenesis by downregulating BTF3L4, thereby impeding tumor growth.^[3]^ Dysregulated miRNAs, such as miR-409-3p, also influence epithelial-mesenchymal transition and metastasis via KLF17.^[4]^ These findings emphasize miRNAs as central regulators of GC hallmarks, though their causal roles and clinical translation remain underexplored.

The potential of miRNAs as diagnostic and prognostic biomarkers has gained traction due to their stability in bodily fluids, including plasma.^[5]^ For example, plasma miR-21, miR-25, and miR-223 levels distinguish GC patients from healthy controls,^[6]^ while tissue-specific miRNAs like miR-381-3p target FGFR2 to inhibit proliferation.^[7]^ However, observational studies face limitations, including confounding factors and reverse causation, which obscure causal inference. Competing endogenous RNA (ceRNA) networks, such as the lncRNA-miRNA-mRNA axis involving PVT1/miR-130a-3p/RECK, further complicate interpretations.^[8]^ Although these studies highlight miRNA-mediated regulatory networks, their reliance on correlational data limits clinical applicability. Robust methodologies are needed to establish causality and prioritize therapeutic targets.

Mendelian randomization (MR) has emerged as a powerful tool to infer causality by leveraging genetic variants as instrumental variables (IVs) to mitigate confounding. This approach has been applied successfully in oncology; for example, MR analysis linked miRNA-associated SNPs to lung cancer risk.^[9]^ In GC, MR could address critical gaps, such as disentangling whether miRNA dysregulation drives carcinogenesis or merely reflects disease progression. Previous GC studies identified miRNA-related SNPs (e.g., rs11614913 in miR-196a2) associated with survival,^[10]^ but causal relationships remain unexplored. MR’s ability to utilize expression quantitative trait loci (eQTLs) from plasma miRNAs offers unique advantages, as plasma miRNAs are noninvasive biomarkers with clinical relevance.^[11]^

This study employs a 2-sample MR framework to investigate causal relationships between plasma circulatory miRNAs and GC risk using independent eQTL datasets. By integrating genome-wide association studies (GWAS) and miRNA-eQTL data, we address key limitations of prior research, such as tissue specificity and confounding. By applying MR, we aim to prioritize miRNAs with causal roles in GC, offering insights into novel therapeutic avenues and biomarkers. This study bridges molecular biology and epidemiology, advancing precision oncology in GC management.

## 2. Materials and methods

### 2.1. Data sources and preprocessing

Plasma miRNA-eQTL data were obtained from the cohort reported by Huan et al,^[11]^ comprising 280 high-quality miRNAs and approximately 10 million associated SNPs. To mitigate potential pleiotropic effects, we focused exclusively on cis-acting miRNA-eQTLs and further excluded SNPs located in coding regions with synonymous or missense consequences. SNPs with a Benjamini-Hochberg adjusted false discovery rate (FDR) < 0.1 were retained as IVs.

Transcriptomic profiles, somatic mutation data, and clinical follow-up data for the TCGA-STAD cohort were retrieved from the cancer genome atlas (TCGA), including 36 normal samples and 412 GC cases. Additionally, GWAS summary statistics for GC were acquired from the IEU OpenGWAS Project (https://gwas.mrcieu.ac.uk/) under accession IDs ebi-a-GCST90018849, bbj-a-119, and ebi-a-GCST90018629. Detailed metadata for these datasets are summarized in Table 1.

**Table 1:** Summary of gastric cancer GWAS datasets.

GWAS ID	Year	Ethnicity	Number of case	Number of control	Number of SNPs	Author
ebi-a-GCST90018849	2021	European	1029	475,087	24,188,662	Sakaue S
bbj-a-119	2019	East Asian	6563	195,745	8885,324	Ishigaki K
ebi-a-GCST90018629	2021	East Asian	7921	159,201	12,453,108	Sakaue S

GWAS = genome-wide association studies.

### 2.2. Two-Sample MR analysis

Genetic associations between miRNAs and GC were assessed using the TwoSampleMR package (v0.6.8) in R. Independent IVs were identified through linkage disequilibrium clumping (*r*^2^ < 0.5, 10 kb window). SNPs showing genome-wide significance (*P* ≤5 × 10^−8^) for GC in GWAS data were excluded. Exposure and outcome datasets were harmonized to align effect alleles, and palindromic SNPs were removed. Causal relationships were established if the following criteria were met: significant association in inverse-variance weighted (IVW) analysis (*P* <.05, FDR < 0.1); presence of ≥ 3 SNPs in MR testing; concordant effect directions between IVW and MR-Egger estimates; and consistent IVW trends across all GC GWAS datasets. Horizontal pleiotropy was evaluated via MR-Egger regression, while leave-one-out sensitivity analyses assessed the robustness of causal estimates to individual SNP exclusion.

### 2.3. miRNA-target prediction and functional enrichment

Experimentally validated miRNA-target interactions were curated from miRTarBase v9.0 using miRNet 2.0 (https://www.mirnet.ca/). Resulting networks were visualized in Cytoscape v3.10.2. Functional enrichment analysis of target genes was performed with the ClusterProfiler package (v4.12.6), covering gene ontology terms and Kyoto encyclopedia of genes and genomes pathways.

### 2.4. Survival and somatic mutation analysis

Differentially expressed genes (DEGs) between tumor and normal tissues in the TCGA-STAD cohort were identified (log_2_ fold change > 1, adjusted *P* <.05). DEGs were subjected to univariate Cox proportional hazards regression to evaluate associations with overall survival (*P* <.05). Somatic mutation landscapes were analyzed using the maftools package. Pan-cancer analysis was performed using the ENCORI Pan-Cancer Analysis Platform (https://rnasysu.com/encori/panCancer.php).

### 2.5. Statistical analysis

All analyses were conducted in R (v4.3.1). Multiple testing corrections employed the Benjamini-Hochberg method (FDR < 0.1). Sensitivity thresholds for differential expression and survival analyses followed established standards for high-throughput genomic studies.

## 3. Results

### 3.1. Identification of serum miRNAs with causal associations to GC risk

**Figure 1.**
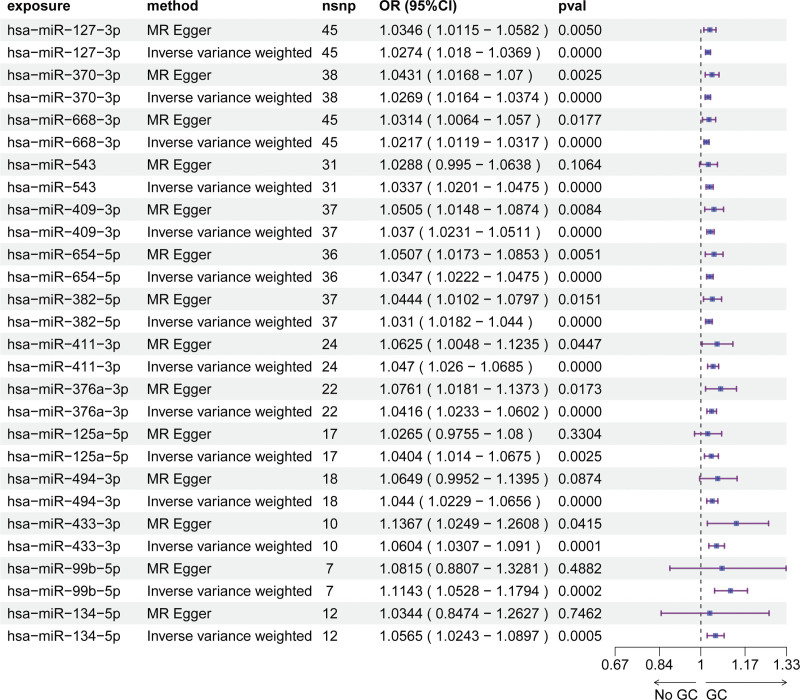
Mendelian randomization analysis of serum miRNAs causally associated with GC risk (ebi-a-GCST90018629 dataset). GC = gastric cancer, miRNAs = microRNAs.

**Figure 2.**
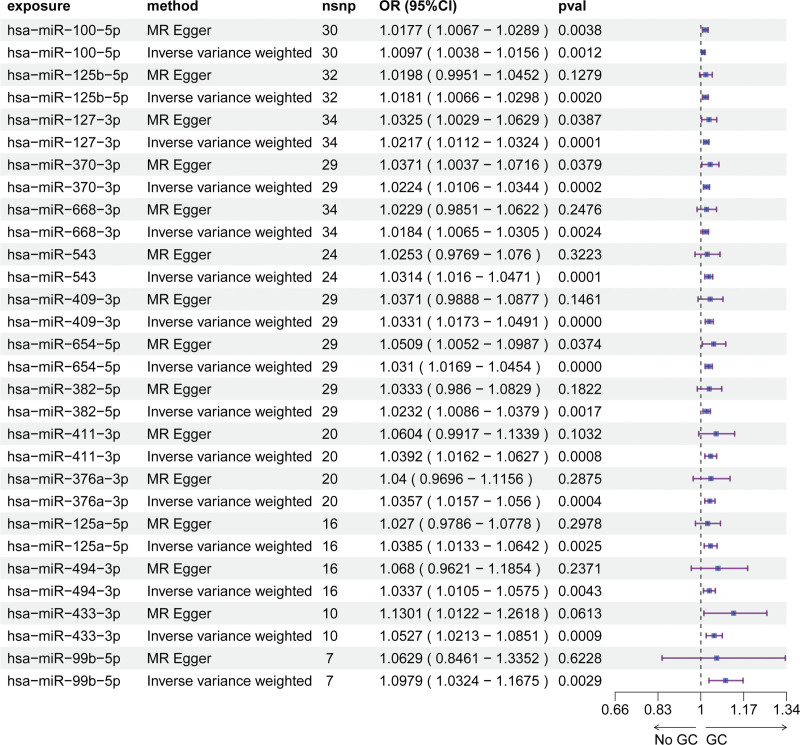
Mendelian randomization analysis of serum miRNAs causally associated with GC risk (ebi-a-GCST90018849 dataset). GC = gastric cancer, miRNAs = microRNAs.

**Figure 3.**
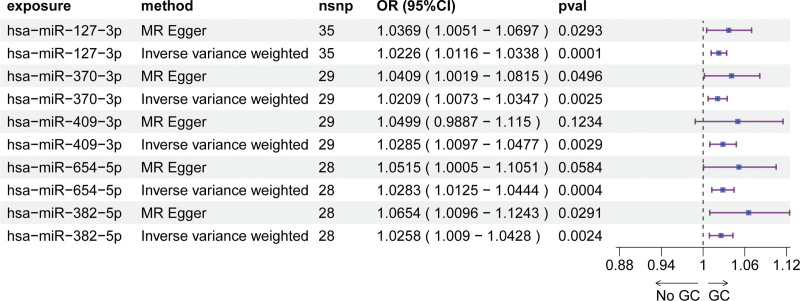
Mendelian randomization analysis of serum miRNAs causally associated with GC risk (bbj-a-119 dataset). GC = gastric cancer, miRNAs = microRNAs.

Using serum miRNA-eQTLs from the Huan et al cohort (9500 SNPs spanning 75 miRNAs), we performed MR analyses to assess causal links between miRNA expression and GC susceptibility (Table S1, Supplemental Digital Content, https://links.lww.com/MD/R72). Across 3 independent GWAS datasets, distinct serum miRNA profiles were associated with GC risk. Specifically, the ebi-a-GCST90018849 dataset implicated 14 miRNAs (IVW *P* <.05, FDR < 0.1; Fig. 1, Table S2, Supplemental Digital Content, https://links.lww.com/MD/R72), while the ebi-a-GCST90018629 dataset identified 15 risk-associated miRNAs (IVW *P* <.05, FDR < 0.1; Fig. 2, Table S3, Supplemental Digital Content, https://links.lww.com/MD/R72). In the bbj-a-119 dataset, 5 miRNAs exhibited significant causal effects on GC risk (IVW *P* <.05; Fig. 3, Table S4, Supplemental Digital Content, https://links.lww.com/MD/R72). MR-Egger regression detected no evidence of horizontal pleiotropy (intercept *P* >.05 for all analyses; Table S5, Supplemental Digital Content, https://links.lww.com/MD/R72), supporting the validity of causal inferences. Leave-one-out sensitivity analyses confirmed that no individual SNP disproportionately drove the observed associations (Fig. S1–3, Supplemental Digital Content, https://links.lww.com/MD/R71), reinforcing the stability of our findings.

### 3.2. Identification of causal miRNAs and their experimentally validated targets

**Figure 4.**
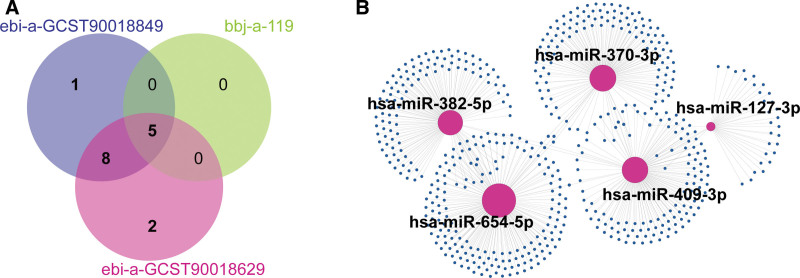
Causal miRNA identification and their targets. (A) Venn diagram illustrating overlap of causal miRNAs across 3 GWAS datasets (IVW *P* < .05, FDR < 0.1). (B) Experimentally validated target gene network (miRTarBase v9.0) for 5 causal miRNAs. Node size reflects targeting frequency; edges indicate miRNA-gene interactions. FDR = false discovery rate, GWAS = genome-wide association studies, IVW = inverse-variance weighted, miRNAs = microRNAs

Intersection of MR results across 3 independent GWAS datasets identified 5 miRNAs exhibiting consistent causal associations with elevated GC risk (IVW *P* <.05, FDR < 0.1 in all cohorts): hsa-miR-127-3p, hsa-miR-370-3p, hsa-miR-382-5p, hsa-miR-409-3p, and hsa-miR-654-5p (Fig. 4A). Experimentally validated target genes for these miRNAs were curated from miRTarBase (v9.0), yielding a total of 549 unique targets. The regulatory landscape revealed distinct targeting capacities: hsa-miR-127-3p (n = 33 targets), hsa-miR-370-3p (n = 126), hsa-miR-382-5p (n = 118), hsa-miR-409-3p (n = 124), and hsa-miR-654-5p (n = 165), suggesting heterogeneous roles in GC pathogenesis (Fig. 4B, Table S6, Supplemental Digital Content, https://links.lww.com/MD/R72).

### 3.3. Functional characterization of miRNA targets in gastric carcinogenesis

**Figure 5.**
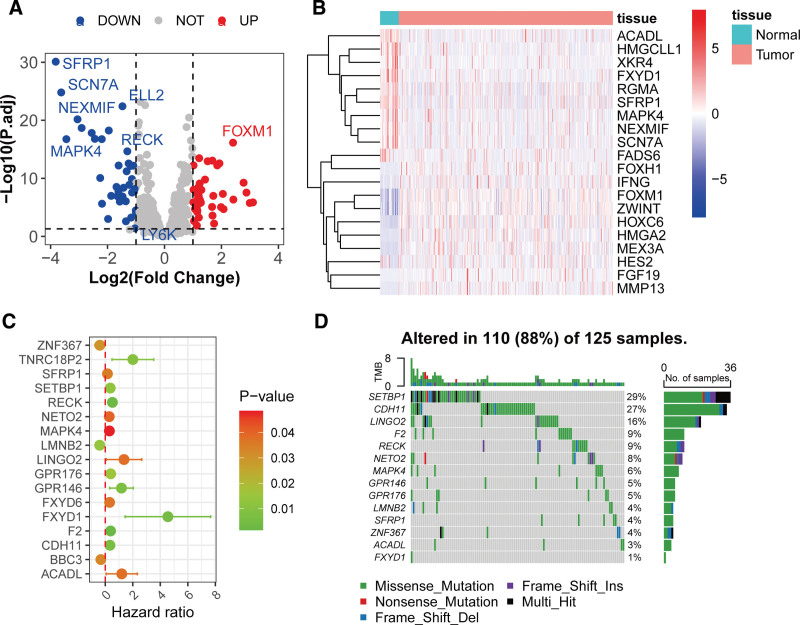
Functional characterization of miRNA targets in gastric carcinogenesis. (A) Volcano plot of DEGs between GC and normal tissues (log2FC > 1, FDR < 0.05). (B) Hierarchical clustering of DEGs (rows: genes; columns: samples). (C) Kaplan–Meier curves for prognostic targets (log-rank *P* < .05). (D) Oncoprint of somatic mutations in prognostic targets. DEGs = differentially expressed targets, FDR = false discovery rate, GC = gastric cancer.

To delineate the mechanistic roles of causal miRNAs in GC, we analyzed their targets for differential expression, prognostic relevance, and mutational patterns. Comparative analysis in the TCGA-STAD cohort identified 76 significantly dysregulated targets (39 upregulated, 37 downregulated; log_2_ fold change > 1, adjusted *P* <.05) in tumor versus normal tissues (Fig. 5A). A clustered heatmap highlighted distinct expression profiles of select DEGs between tumor and normal tissues (Fig. 5B). Univariate Cox proportional hazards modeling revealed 17 DEGs significantly associated with overall survival (log-rank *P* <.05). Notably, elevated expression of ZNF367 (HR = 0.76, 95% CI = 0.59–0.97), LMNB2 (HR = 0.75, 95% CI = 0.60–0.94), and BBC3 (HR = 0.80, 95% CI = 0.59–0.97) predicted improved prognosis, whereas 14 genes correlated with poorer survival (HR < 1; Fig. 5C, Table S7, Supplemental Digital Content, https://links.lww.com/MD/R72). Somatic mutation analysis using maftools demonstrated that 14 prognostic targets harbored nonsynonymous mutations (Fig. 5D). SETBP1 exhibited the highest mutation frequency (29%), followed by CDH11 (27%) and LINGO2 (16%).

### 3.4. Pathway and biological process enrichment of prognostic miRNA targets

**Figure 6.**
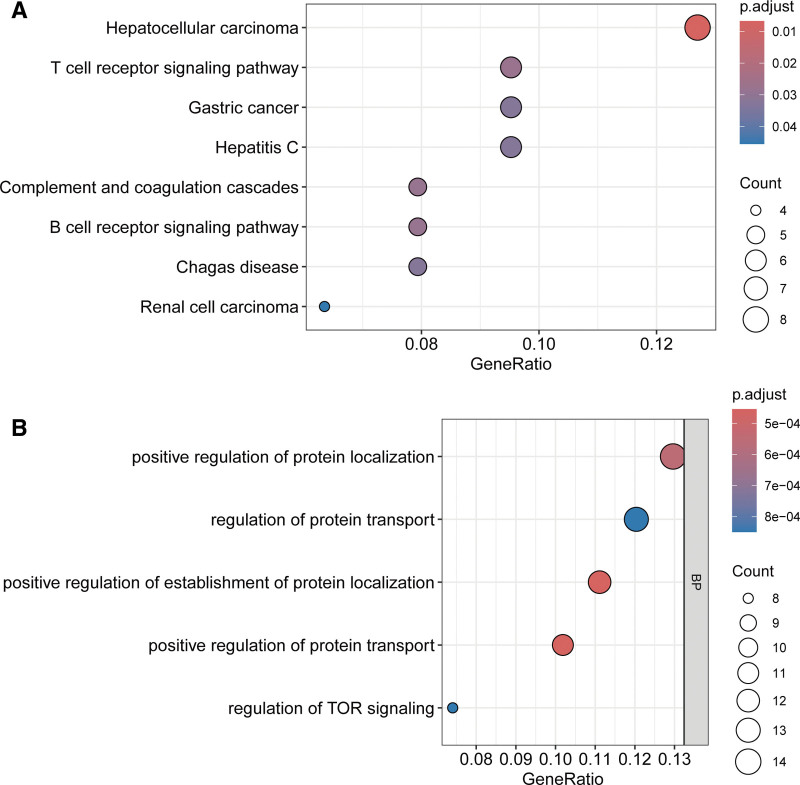
Pathway enrichment analysis of miRNA targets. (A) KEGG pathways enriched in prognostic targets. (B) GO biological processes modulated by causal miRNAs. GO = gene ontology, KEGG = Kyoto encyclopedia of genes and genomes, miRNAs = microRNAs.

To elucidate the biological significance of prognosis-associated miRNA targets, we conducted pathway and ontology enrichment analyses. Kyoto encyclopedia of genes and genomes pathway analysis (FDR < 0.05) identified significant overrepresentation in cancer-related pathways, including hepatocellular carcinoma, gastric cancer, and renal cell carcinoma, alongside immune regulatory pathways such as T cell receptor signaling and B cell receptor signaling, as well as complement and coagulation cascades (Fig. 6A). Gene ontology analysis of biological processes highlighted enrichment in terms governing intracellular trafficking and growth signaling: positive regulation of protein localization, regulation of protein transport, and regulation of TOR signaling, a key modulator of cell proliferation and survival (Fig. 6B).

### 3.5. Pan-cancer analysis of causal miRNA expression and prognostic significance

**Figure 7.**
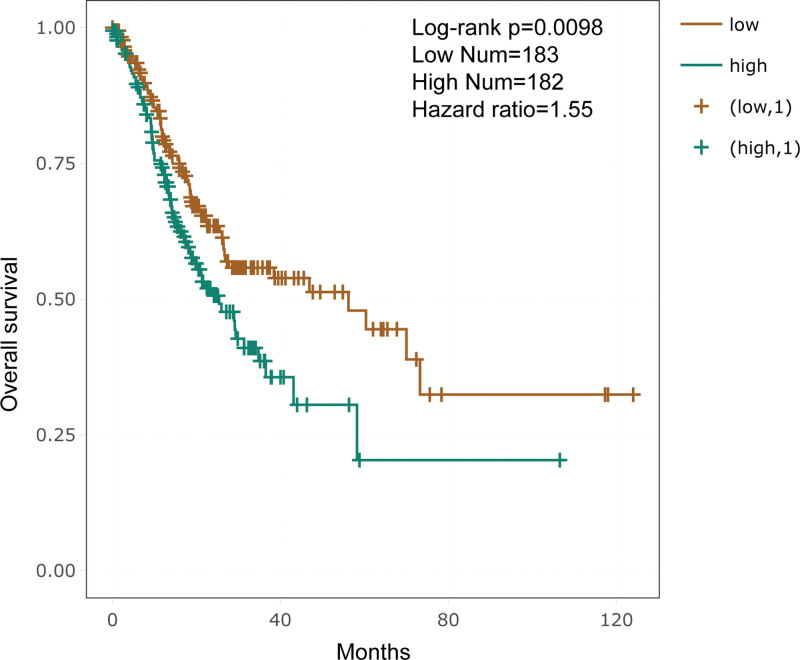
Kaplan–Meier survival curves comparing overall survival between gastric cancer patients with high and low hsa-miR-409-3p expression. Patients with low expression exhibited significantly improved survival (HR = 1.55, log-rank *P* = .0098).

To delineate the oncogenic roles of causal miRNAs across malignancies, we systematically analyzed their expression and prognostic relevance using the ENCORI Pan-Cancer Analysis Platform. Our pan-cancer screening identified 5 causal miRNAs with significant dysregulation in 16 cancer types (Table S8, Supplemental Digital Content, https://links.lww.com/MD/R72). In GC, 4 miRNAs (hsa-miR-370-3p, hsa-miR-409-3p, hsa-miR-654-5p, and hsa-miR-382-5p) were markedly upregulated, while hsa-miR-127-3p showed no significant alteration (Table 2). Survival analysis revealed that elevated expression of these 5 miRNAs predicted adverse outcomes (HR > 1) in 7 cancers, underscoring their broad oncogenic potential (Table S9, Supplemental Digital Content, https://links.lww.com/MD/R72). Strikingly, in GC, only hsa-miR-409-3p emerged as an independent prognostic biomarker (Table 3). Kaplan–Meier survival analysis revealed that patients with GC exhibiting low hsa-miR-409-3p expression had significantly better overall survival compared with those with high expression (HR = 1.55, log‑rank *P* = .0098; Fig. 7). Mechanistically, co-expression analysis demonstrated an inverse regulatory relationship between hsa-miR-409-3p and 7 GC-associated targets, including XKR4, F2, ATAD5, GNAL, GDNF, UNC13A, and ELL2 (Table 4).

**Table 2:** Differential expression of causal miRNAs in GC.

miRNAs	Fold Change	*P*-value	FDR
hsa-miR-127-3p	1.37	.1	0.34
**hsa-miR-370-3p**	**1.84**	**.0019**	**0.012**
**hsa-miR-409-3p**	**2.09**	**1.60 × 10^−08^**	**2.90 × 10^−07^**
**hsa-miR-654-5p**	**2.17**	**0.0033**	**0.019**
**hsa-miR-382-5p**	**2.06**	**3.60 × 10^−08^**	**6.30 × 10^−07^**

Bold values indicate *P*-values that are statistically significant (*P* < .05).

FDR = false discovery rate, GC = gastric cancer, miRNAs = microRNAs.

**Table 3:** Prognostic significance of causal miRNAs in GC.

miRNAs	CancerNum	Median	Coefficient	HR	*P*-value
hsa-miR-127-3p	365	472.68	0.1	1.1	.56
hsa-miR-370-3p	365	4.14	0.3	1.35	.076
**hsa-miR-409-3p**	**365**	**18.48**	**0.44**	**1.55**	**.0098**
hsa-miR-654-5p	365	1.15	0.03	1.03	.88
hsa-miR-382-5p	365	15.33	0.26	1.3	.12

Bold values indicate *P*-values that are statistically significant (*P* < .05).

GC = gastric cancer, miRNAs = microRNAs.

**Table 4:** Co-expression analysis of hsa-miR-409-3p and target genes differentially expressed in GC.

Gene symbol	Sample number	Coefficient-R	*P*-value
XKR4	372	−0.142	6.06 × 10^−03^
F2	372	−0.068	1.89 × 10^−01^
ATAD5	372	−0.061	2.39 × 10^−01^
GNAL	372	−0.06	2.51 × 10^−01^
GDNF	372	−0.055	2.91 × 10^−01^
UNC13A	372	−0.043	4.07 × 10^−01^
ELL2	372	−0.024	6.38 × 10^−01^
MET	372	0.013	7.96 × 10^−01^
CDCP1	372	0.017	7.38 × 10^−01^
IFNG	372	0.029	5.76 × 10^−01^
UGT2B17	372	0.029	5.81 × 10^−01^
ZNF367	372	0.055	2.87 × 10^−01^
LHX2	372	0.078	1.35 × 10^−01^
GCK	372	0.104	4.59 × 10^−02^
C1QTNF3	372	0.148	4.16 × 10^−03^
LVRN	372	0.154	2.99 × 10^−03^
RECK	372	0.183	4.00 × 10^−04^
IGFBP3	372	0.227	9.50 × 10^−06^

GC = gastric cancer.

## 4. Discussion

This study represents the first comprehensive MR analysis to establish causal relationships between plasma circulatory miRNAs and GC risk. By integrating miRNA-eQTL data with 3 independent GWAS datasets, we identified 5 miRNAs (hsa-miR-127-3p, hsa-miR-370-3p, hsa-miR-382-5p, hsa-miR-409-3p, and hsa-miR-654-5p) as robust causal regulators of GC susceptibility. These findings advance our understanding of miRNA-driven oncogenesis and provide a framework for prioritizing therapeutic targets and noninvasive biomarkers.

Our MR analysis provides novel genetic evidence that elevated levels of hsa-miR-409-3p are causally associated with an increased risk of gastric cancer. This causal estimate was consistent across multiple datasets and robust to sensitivity analyses. This pro-oncogenic role is supported by several experimental studies. For example, Cheng et al (2025) revealed that the lncRNA SNHG4 promotes GC progression by sponging miR-409-3p, thereby derepressing its target CREB1.^[12]^ We further validated this inverse co-expression relationship between miR-409-3p and CREB1 in the TCGA-STAD cohort, lending functional credence to our genetic findings. However, our results appear to contrast with a study by Feng et al. (2021), which reported that hyperthermia-induced miR-409-3p overexpression suppressed GC metastasis by targeting KLF17.^[4]^ This apparent paradox may reflect context-dependent roles of miR-409-3p, where its tumor-suppressive effects are hijacked by oncogenic ceRNA networks in advanced GC. Notably, our pan-cancer analysis identified miR-409-3p as an independent prognostic biomarker in GC, consistent with its regulation of multiple oncogenic pathways. Experimental studies have validated miR-409-3p targets including PHF10,^[13]^ MAP7,^[14]^ and RDX,^[15]^ which collectively modulate proliferation, apoptosis, and cytoskeletal remodeling. The MR-derived association between miR-409-3p and somatic mutations in SETBP1/CDH11 further suggests its involvement in genomic instability, a hallmark of GC progression. Notwithstanding these complex mechanisms, our study uniquely establishes the causal, rather than merely correlative, link between miR-409-3p and GC susceptibility. Its identification as an independent prognostic biomarker in our pan-cancer analysis further highlights its clinical relevance specific to GC.

Intriguingly, our MR analysis suggested a putative causal role for hsa-miR-127-3p in increasing GC risk, a finding that stands in contrast to its well-characterized tumor-suppressive functions in other solid tumors. Indeed, extensive literature supports miR-127-3p’s role as a tumor suppressor. For instance, in ovarian cancer, it inhibits proliferation by targeting MAPK4.^[16]^ while in prostate cancer, its transcriptional downregulation promotes bone metastasis.^[17]^ Similarly, in oral squamous cell carcinoma, it targets KIF3B to inhibit development.^[18]^ The discrepancy between our genetic evidence and these functional studies highlights the critical difference between tissue-specific miRNA activity and systemic, genetically determined miRNA levels measured in plasma. It is plausible that the observed elevation of plasma miR-127-3p in at-risk individuals represents a compensatory antitumor response or a consequence of tumor-derived exosomal packaging, rather than direct driver activity. Our study is among the first to genetically implicate circulating miR-127-3p in oncogenesis, suggesting its role may be more complex and context-dependent than previously appreciated. This novel hypothesis warrants further investigation.

Our MR findings established hsa-miR-370-3p as a causal risk factor for GC, which is consistent with its documented oncogenic potential in certain contexts. The role of miR-370-3p in cancer is complex and tissue-specific, and our results add to a body of evidence showing its capacity to drive malignancy. For example, elevated plasma miR-370-3p levels correlate with poor prognosis in pancreatic ductal adenocarcinoma,^[19]^ and in breast cancer, it can promote progression via the FBLN5/NF-κB axis.^[20]^ It is important to note that miR-370-3p can also exert tumor-suppressive effects, as seen in nonfunctional pituitary adenomas where it targets HMGA2,^[21]^ and in glioblastoma where it suppresses stem-like cell malignancy.^[22]^ This functional duality underscores that the ultimate effect of a miRNA is dictated by its specific target repertoire within a given cellular microenvironment. Our genetic approach, which infers the net effect of lifelong perturbation, robustly indicates that the overall systemic impact of miR-370-3p in GC pathogenesis is causally oncogenic.

We identified hsa-miR-382-5p as a novel causal mediator of GC susceptibility in our MR framework. This finding is mechanistically supported by studies demonstrating its oncogenic functions through key pathways. For instance, in acute promyelocytic leukemia and hepatocellular carcinoma, miR-382-5p promotes oncogenesis by targeting PTEN, thereby activating the PI3K/Akt signaling pathway.^[23,24]^ Furthermore, in oral squamous cell carcinoma, cancer-associated fibroblast-derived exosomal miR-382-5p facilitates metastasis,^[25]^ highlighting a potential paracrine mechanism that could be relevant to GC. Notwithstanding these evidence, miR-382-5p has also been reported to act as a tumor suppressor in glioma.^[26]^ This reinforces the principle of context-dependency. The consistent causal effect observed in our MR analysis suggests that the PTEN-mediated oncogenic axis is likely the dominant pathway influenced by circulating miR-382-5p in the context of GC development. Therefore, our study not only reveals a new causal role for miR-382-5p in GC but also provides genetic evidence that prioritizes its oncogenic function over its suppressive role in this specific malignancy.

Our MR analysis provides novel genetic evidence that genetically elevated levels of hsa-miR-654-5p are causally linked to an increased risk of gastric cancer. This positions it as a potential oncogenic driver in the stomach microenvironment. This pro-tumorigenic role is strongly supported by existing functional studies specific to GC. Zhou et al. (2021) demonstrated that miR-654-5p promotes gastric cancer progression by activating the NF-κB pathway via direct suppression of GPRIN1.^[27]^ Our results genetically validate this earlier mechanistic finding, moving beyond correlation to establish a causal relationship. The oncogenic function of miR-654-5p, however, is not universal and exhibits a striking tissue-specific duality. In contrast to its role in GC, miR-654-5p acts as a tumor suppressor in colorectal cancer (CRC), where its downregulation is associated with poor prognosis and it functions by targeting HAX-1.^[28–30]^ It also shows oncogenic properties in oral squamous cell carcinoma via the Ras/MAPK pathway.^[31]^ This functional dichotomy is likely governed by the distinct landscape of target genes expressed in different tissues. Our study, through the MR framework, captures the net causal effect of miR-654-5p perturbation within the context of GC etiology. Despite its suppressive role in CRC, our findings unequivocally demonstrate that its systemic influence on GC risk is causally oncogenic. This underscores the necessity of evaluating miRNA functions in a disease-specific manner and highlights the value of our MR approach in delineating these complex relationships.

Our multi-omics approach revealed that causal miRNAs converge on critical cancer hallmarks through pleiotropic target regulation. The enrichment of miRNA targets in T/B cell receptor signaling and complement cascades highlights the interplay between miRNA dysregulation and tumor immune evasion – a finding consistent with recent studies linking plasma miRNAs to immunosuppressive microenvironments in GC.^[32–34]^ The prognostic significance of targets like ZNF367 and BBC3, which regulate genomic stability and apoptosis,^[35,36]^ underscores the therapeutic potential of restoring miRNA-mediated tumor suppressor networks. Furthermore, the high mutation frequency observed in SETBP1 and CDH11 suggests synergistic effects between somatic alterations and miRNA dysregulation in driving GC progression.^[37–39]^ We also examined the co-expression patterns between miR-409-3p and its target genes in GC. It is noteworthy that correlation coefficients for some targets did not reach conventional thresholds of statistical significance (*P* >.2). This is not altogether surprising for miRNA–mRNA interactions analyzed in bulk tumor tissues, where the effects of individual miRNAs are often subtle, cumulative, and susceptible to masking by tumor heterogeneity, posttranscriptional regulation, and asynchrony between miRNA expression and target degradation.^[40–42]^

Methodologically, our study addresses critical limitations of prior observational research by employing MR to minimize confounding and reverse causation. The consistency of causal effects across diverse populations (European and East Asian GWAS datasets) strengthens the generalizability of our findings. The use of plasma-derived eQTLs provides distinct advantages over tissue-specific analyses, as circulatory miRNAs reflect systemic intercellular communication and are more feasible for clinical translation.^[43]^ However, certain limitations warrant consideration. First, restricting instruments to cis-eQTLs may have excluded trans-regulatory SNPs with pleiotropic effects on miRNA biogenesis. Second, while plasma miRNAs offer noninvasive diagnostic potential, their expression levels may not fully mirror tissue-specific profiles due to selective miRNA packaging into extracellular vesicles. Third, despite the absence of significant horizontal pleiotropy as indicated by MR-Egger regression (intercept *P* >.05) and the robustness of results in leave-one-out sensitivity analyses, residual confounding may still pose a threat to causal inference. For instance, certain genetic variants might affect gastric cancer risk through pathways unrelated to miRNA biology – such as inflammation, metabolism, or immune regulation – thereby introducing bias.^[44]^ Moreover, since the eQTL data were derived from plasma miRNA, their expression could be influenced by systemic physiological states (e.g., inflammation, hepatic or renal function), which may themselves correlate with gastric cancer risk, constituting a potential source of confounding.^[45,46]^ Additionally, the relatively limited sample size of the plasma miRNA-eQTL dataset (n = 5239) compared to the GWAS cohorts may constrain statistical power to identify weaker IVs and limit the generalizability of the results. Future investigations leveraging larger eQTL resources are needed to validate these findings.

Future studies should prioritize functional validation of identified miRNAs using in vivo models and single-cell sequencing to delineate cell-type-specific regulatory mechanisms. The lack of association between miR-127-3p expression and survival despite its causal role suggests context-dependent functions that may involve stromal interactions or epigenetic modifications. Additionally, expanding MR analyses to include miRNA processing genes (e.g., DROSHA, XPO5) could uncover upstream regulators of the identified miRNAs. Clinically, longitudinal studies are needed to evaluate the dynamic changes of causal miRNAs during GC progression and their predictive value for therapeutic response.

## 5. Conclusion

In conclusion, this MR study establishes a causal repertoire of plasma miRNAs in gastric carcinogenesis and provides mechanistic insights into their roles as oncogenic drivers and prognostic indicators. By bridging genetic epidemiology with functional genomics, our findings lay the groundwork for developing miRNA-based precision strategies in GC prevention and treatment.

## Author contributions

**Conceptualization:** Yejue Lin, Ming Luo.

**Data curation:** Yejue Lin, Ming Luo.

**Formal analysis:** Yejue Lin, Ming Luo.

**Funding acquisition:** Yejue Lin, Ming Luo.

**Investigation:** Yejue Lin, Ming Luo.

**Methodology:** Yejue Lin, Ming Luo.

**Project administration:** Yejue Lin, Ming Luo.

**Resources:** Yejue Lin, Ming Luo.

**Software:** Yejue Lin, Ming Luo.

**Supervision:** Yejue Lin, Ming Luo.

**Validation:** Yejue Lin, Ming Luo.

**Visualization:** Yejue Lin, Ming Luo.

**Writing – original draft:** Yejue Lin, Ming Luo.

**Writing – review & editing:** Yejue Lin, Ming Luo.

## Supplementary Material





## References

[1] Thrift AP Wenker TN El-Serag HB. Global burden of gastric cancer: epidemiological trends, risk factors, screening and prevention. Nat Rev Clin Oncol. 2023;20:338–49. 36959359 10.1038/s41571-023-00747-0

[2] Bai HX Qiu X-M Xu C-H Guo J-Q. MiRNA-145-5p inhibits gastric cancer progression via the serpin family E member 1- extracellular signal-regulated kinase-1/2 axis. World J Gastrointest Oncol. 2024;16:2123–40. 38764835 10.4251/wjgo.v16.i5.2123 PMC11099451

[3] Cao H Wang Z Guo Q Qin S Li D. MIR194-2HG, a miRNA host gene activated by HNF4A, inhibits gastric cancer by regulating microRNA biogenesis. Biol Direct. 2024;19:95. 39425187 10.1186/s13062-024-00549-z PMC11487860

[4] Feng J Li K Liu G Feng Y Shi H Zhang X. Precision hyperthermia-induced miRNA-409-3p upregulation inhibits migration, invasion, and EMT of gastric cancer cells by targeting KLF17. Biochem Biophys Res Commun. 2021;549:113–9. 33667708 10.1016/j.bbrc.2021.02.063

[5] Cuellar-Gomez H Ocharán-Hernández MªE Calzada-Mendoza CC Comoto-Santacruz DA. Serum miRNA profile as a potential tool for non-invasive gastric cancer diagnosis in Mexican patients. Cir Cir. 2021;89:748–54. 34851581 10.24875/CIRU.20000963

[6] Gao X Liu H Wu Q. miRNA-381-3p functions as a tumor suppressor to inhibit gastric cancer by targeting fibroblast growth factor receptor-2. Cancer Biother Radiopharm. 2023;38:396–404. 35029520 10.1089/cbr.2021.0357

[7] Deng C Peng J Yuan C. Comprehensive analysis to construct a novel immune-related prognostic panel in aging-related gastric cancer based on the lncRNA–miRNA-mRNA ceRNA network. Front Mol Biosci. 2023;10:1163977. 37255541 10.3389/fmolb.2023.1163977 PMC10226425

[8] Huang R Cho WC Sun Y Katie Chan KH. The lung cancer associated MicroRNAs and single nucleotides polymorphisms: a mendelian randomization analysis. Annu Int Conf IEEE Eng Med Biol Soc. 2020;2020:2346–52. 33018478 10.1109/EMBC44109.2020.9176344

[9] Chen ZZ Wang W-P Xue H-M Liang Y. The lncRNA-miRNA-integrin alpha V ceRNA network can affect the occurrence and prognosis of gastric cancer. Int J Clin Exp Pathol. 2022;15:388–402. 36381423 PMC9638841

[10] Gilani N Arabi Belaghi R Aftabi Y Faramarzi E Edgünlü T Somi MH. Identifying potential mirna biomarkers for gastric cancer diagnosis using machine learning variable selection approach. Front Genet. 2021;12:779455. 35082831 10.3389/fgene.2021.779455 PMC8785967

[11] Huan T Rong J Liu C. Genome-wide identification of microRNA expression quantitative trait loci. Nat Commun. 2015;6:6601. 25791433 10.1038/ncomms7601 PMC4369777

[12] Cheng Z Hua Y Cao Y Qin J. lncRNA SNHG4 enhanced gastric cancer progression by modulating miR-409-3p/CREB1 axis. Oncol Res. 2025;33:185–98. 39735673 10.32604/or.2024.042281 PMC11671409

[13] Li C Nie H Wang M. MicroRNA-409-3p regulates cell proliferation and apoptosis by targeting PHF10 in gastric cancer. Cancer Lett. 2012;320:189–97. 22388101 10.1016/j.canlet.2012.02.030

[14] Yu L Xie J Liu X Yu Y Wang S. Plasma exosomal CircNEK9 accelerates the progression of gastric cancer via miR-409-3p/MAP7 axis. Dig Dis Sci. 2021;66:4274–89. 33449227 10.1007/s10620-020-06816-z

[15] Zheng B Liang L Huang S. MicroRNA-409 suppresses tumour cell invasion and metastasis by directly targeting radixin in gastric cancers. Oncogene. 2012;31:4509–16. 22179828 10.1038/onc.2011.581

[16] Du SY Huang X-X Li N-M. MiR-127-3p inhibits proliferation of ovarian cancer in rats through down-regulating MAPK4. Eur Rev Med Pharmacol Sci. 2020;24:10383–90. 33155194 10.26355/eurrev_202010_23388

[17] Fan J Du W Zhang H. Transcriptional downregulation of miR-127-3p by CTCF promotes prostate cancer bone metastasis by targeting PSMB5. FEBS Lett. 2020;594:466–76. 31562641 10.1002/1873-3468.13624

[18] Ji L Zhu Z-N He C-J Shen X. MiR-127-3p targets KIF3B to inhibit the development of oral squamous cell carcinoma. Eur Rev Med Pharmacol Sci. 2019;23:630–40. 30720171 10.26355/eurrev_201901_16877

[19] Harada T Uemura K Sumiyoshi T. Increased plasma miR-370-3p expression in poor-outcome patients with pancreatic ductal adenocarcinoma. Pancreatology. 2023;23:996–1002. 37945497 10.1016/j.pan.2023.10.019

[20] Mao J Wang L Wu J. miR-370-3p as a novel biomarker promotes breast cancer progression by targeting FBLN5. Stem Cells Int. 2021;2021:4649890. 34475958 10.1155/2021/4649890 PMC8407987

[21] Cai F Dai C Chen S. CXCL12-regulated miR-370-3p functions as a tumor suppressor gene by targeting HMGA2 in nonfunctional pituitary adenomas. Mol Cell Endocrinol. 2019;488:25–35. 30853598 10.1016/j.mce.2019.02.020

[22] Lulli V Buccarelli M Ilari R. Mir-370-3p impairs glioblastoma stem-like cell malignancy regulating a complex interplay between HMGA2/HIF1A and the oncogenic long non-coding RNA (lncRNA) NEAT1. Int J Mol Sci. 2020;21:3610. 32443824 10.3390/ijms21103610 PMC7279259

[23] Liu D Zhong L Yuan Z. miR-382-5p modulates the ATRA-induced differentiation of acute promyelocytic leukemia by targeting tumor suppressor PTEN. Cell Signal. 2019;54:1–9. 30453015 10.1016/j.cellsig.2018.11.012

[24] Lv B Liu X Zhu X Huang M. miR-382-5p promotes cell invasion in hepatocellular carcinoma by targeting PTEN to activate PI3K/Akt signaling pathway. World J Surg Oncol. 2022;20:175. 35655254 10.1186/s12957-022-02638-7 PMC9161500

[25] Sun LP Xu K Cui J. Cancer‑associated fibroblast‑derived exosomal miR‑382‑5p promotes the migration and invasion of oral squamous cell carcinoma. Oncol Rep. 2019;42:1319–28. 31364748 10.3892/or.2019.7255 PMC6718099

[26] Wang J Chen C Yan X Wang P. The role of miR-382-5p in glioma cell proliferation, migration and invasion. Onco Targets Ther. 2019;12:4993–5002. 31417288 10.2147/OTT.S196322 PMC6601051

[27] Zhou W Li P Jin P. miR-654-5p promotes gastric cancer progression via the GPRIN1/NF-κB pathway. Open Med (Wars). 2021;16:1683–95. 34805531 10.1515/med-2021-0369 PMC8578810

[28] Li P Cai J-X Han F. Expression and significance of miR-654-5p and miR-376b-3p in patients with colon cancer. World J Gastrointest Oncol. 2020;12:492–502. 32368326 10.4251/wjgo.v12.i4.492 PMC7191333

[29] Huang F Wu X Wei M. miR-654-5p Targets HAX-1 to regulate the malignancy behaviors of colorectal cancer cells. Biomed Res Int. 2020;2020:4914707. 32104694 10.1155/2020/4914707 PMC7035500

[30] Du G Ren C Wang J Ma J. The clinical value of blood miR-654-5p, miR-126, miR-10b, and miR-144 in the diagnosis of colorectal cancer. Comput Math Methods Med. 2022;2022:8225966. 36277010 10.1155/2022/8225966 PMC9584656

[31] Lu M Wang C Chen W Mao C Wang J. miR-654-5p targets GRAP to promote proliferation, metastasis, and chemoresistance of oral squamous cell carcinoma through Ras/MAPK signaling. DNA Cell Biol. 2018;37:381–8. 29364705 10.1089/dna.2017.4095

[32] Zhang Y Huo W Sun L. Targeting miR-148b-5p inhibits immunity microenvironment and gastric cancer progression. Front Immunol. 2021;12:590447. 33717068 10.3389/fimmu.2021.590447 PMC7944991

[33] Yong H Fu J Gao G Shi H Zheng D Zhou X. MiR-34a suppresses the proliferation and invasion of gastric cancer by modulating PDL1 in the immune microenvironment. Mol Cell Probes. 2020;53:101601. 32445780 10.1016/j.mcp.2020.101601

[34] Chen J Chen JG Sun B Wu JH Du CY. Integrative analysis of immune microenvironment-related CeRNA regulatory axis in gastric cancer. Math Biosci Eng. 2020;17:3953–71. 32987562 10.3934/mbe.2020219

[35] Ma B Li J Yang W-K Zhang M-G Xie X-D Bai Z-T. N-trans-feruloyloctopamine wakes up BBC3, DDIT3, CDKN1A, and NOXA signals to accelerate HCC cell apoptosis. Anal Cell Pathol (Amst). 2021;2021:1560307. 34123711 10.1155/2021/1560307 PMC8166497

[36] Kong R Ma Y Li W. Zinc finger protein 367 exerts a cancer-promoting role in small cell lung cancer by influencing the CIT/LATS2/YAP signaling cascade. Toxicol Appl Pharmacol. 2024;489:117005. 38880190 10.1016/j.taap.2024.117005

[37] Wang H Gao Y Qin L. Identification of a novel de novo mutation of SETBP1 and new findings of SETBP1 in tumorgenesis. Orphanet J Rare Dis. 2023;18:107. 37150818 10.1186/s13023-023-02705-6 PMC10165755

[38] Fang F Liu C Li Q Xu R Zhang T Shen X. The role of SETBP1 in gastric cancer: friend or foe. Front Oncol. 2022;12:908943. 35898891 10.3389/fonc.2022.908943 PMC9309353

[39] Chen PF Wang F Nie J-Y. Co-expression network analysis identified CDH11 in association with progression and prognosis in gastric cancer. Onco Targets Ther. 2018;11:6425–36. 30323620 10.2147/OTT.S176511 PMC6174304

[40] Ma F Zhan Y Bartolomé-Cabrero R. Analysis of a miR-148a targetome in B cell central tolerance. Front Immunol. 2022;13:861655. 35634349 10.3389/fimmu.2022.861655 PMC9134011

[41] Diener C Keller A Meese E. The miRNA-target interactions: an underestimated intricacy. Nucleic Acids Res. 2024;52:1544–57. 38033323 10.1093/nar/gkad1142 PMC10899768

[42] Ansari S Verma M. miRNA expression based modulation: a new paradigm for the treatment of chronic myeloid leukemia. Biochim Biophys Acta Rev Cancer. 2025;1880:189366. 40482929 10.1016/j.bbcan.2025.189366

[43] Makarova J Turchinovich A Shkurnikov M Tonevitsky A. Extracellular miRNAs and cell-cell communication: problems and prospects. Trends Biochem Sci. 2021;46:640–51. 33610425 10.1016/j.tibs.2021.01.007

[44] Liabeuf D Oshima M Stange DE Sigal M. Stem cells, helicobacter pylori, and mutational landscape: utility of preclinical models to understand carcinogenesis and to direct management of gastric cancer. Gastroenterology. 2022;162:1067–87. 34942172 10.1053/j.gastro.2021.12.252

[45] Mustafa R Mens MMJ van Hilten A. A comprehensive study of genetic regulation and disease associations of plasma circulatory microRNAs using population-level data. Genome Biol. 2024;25:276. 39434104 10.1186/s13059-024-03420-6 PMC11492503

[46] Shuai Y Zhang X Lavrijssen BDA. Dysregulation of plasma circulating microRNAs in all-cause and cause-specific cancers: the rotterdam study. Biomark Res. 2024;12:83. 39135147 10.1186/s40364-024-00626-5 PMC11321125

