# Supplementary material for: Mendelian randomization identifies circulating miRNAs as causal mediators of gastric cancer susceptibility and survival outcomes
Source: Medicine (Baltimore). 2026 Jan 2;105(1):e46833. doi: 10.1097/MD.0000000000046833 (PMC12778131; doi:10.1097/MD.0000000000046833)

Figure S1. Leave-one-out analysis results of the ebi-a-GCST90018849 dataset.


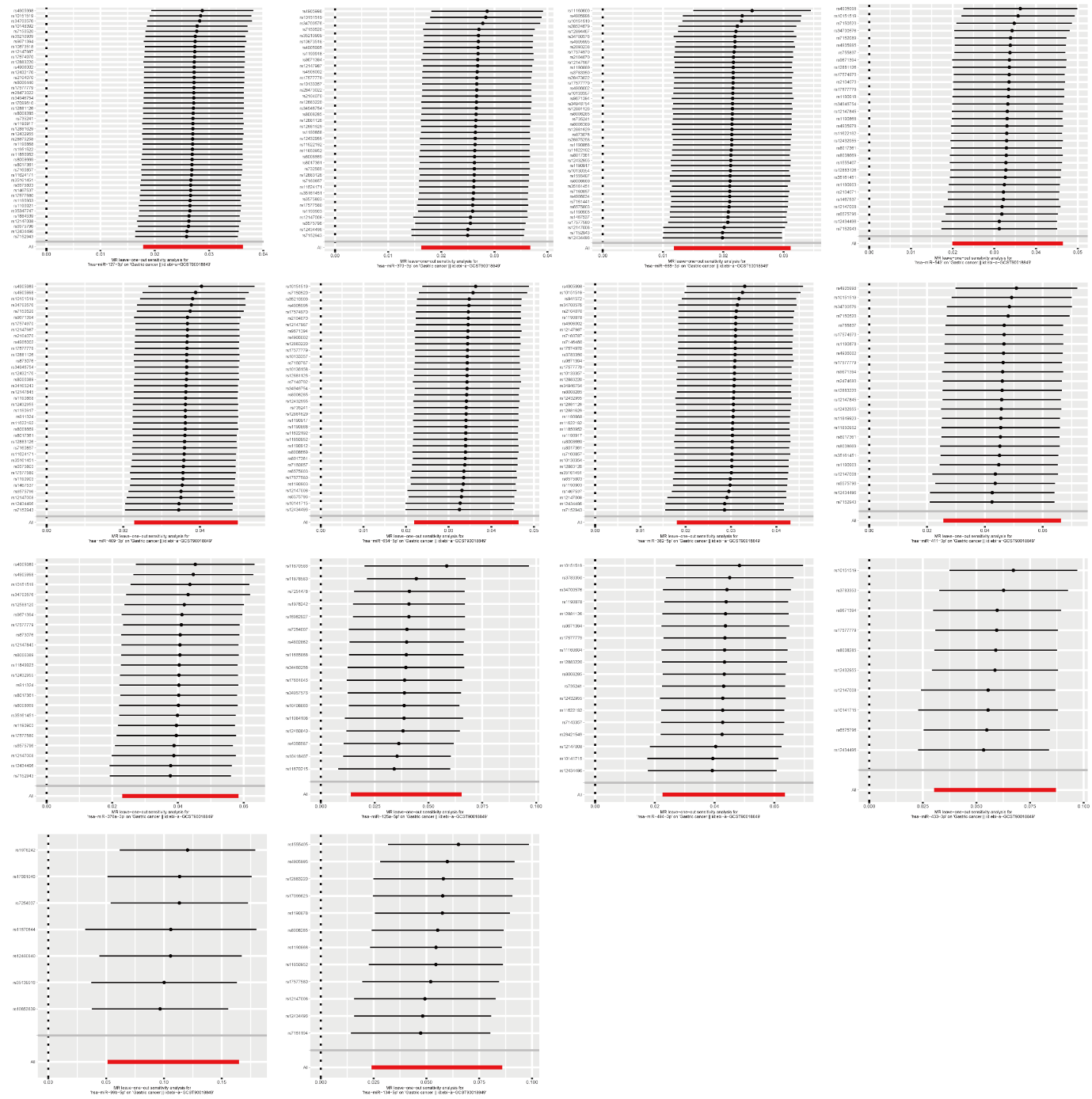


Figure S2. Leave-one-out analysis results of the ebi-a-GCST90018629 dataset.


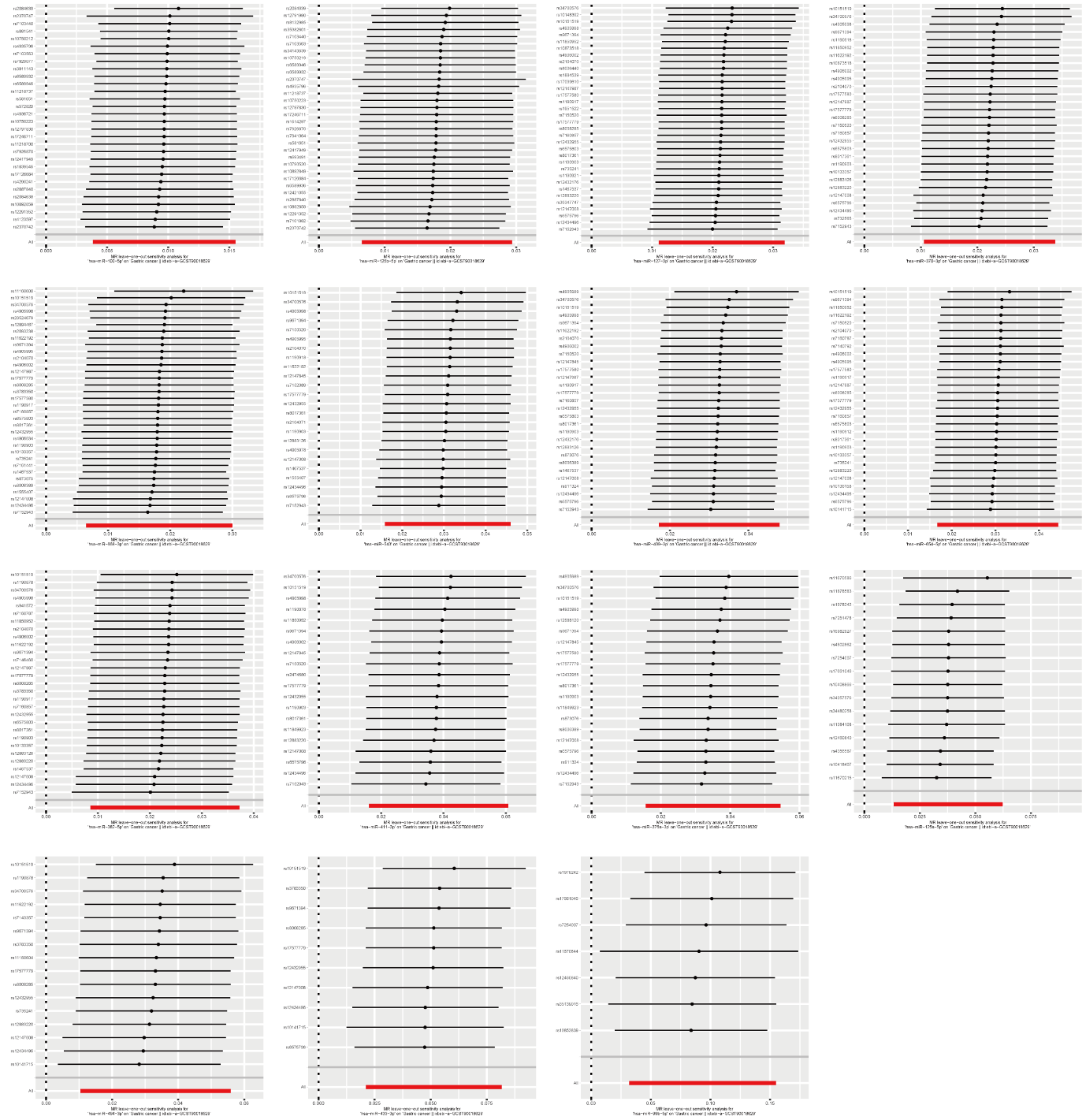


Figure S3. Leave-one-out analysis results of the bbj-a-119 dataset.


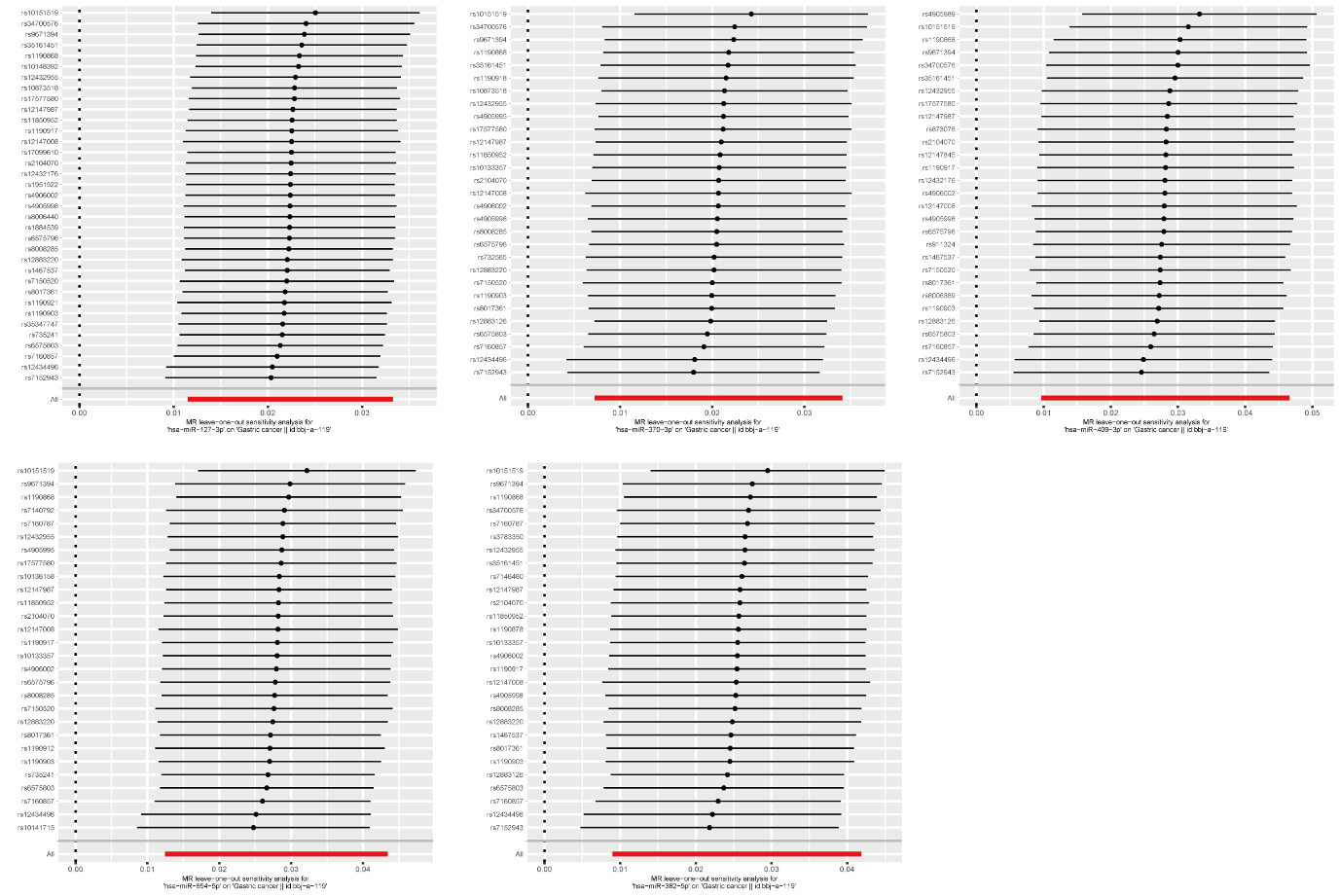

Supplement: Supplementary file 2 [file medi-105-e46833-s002.docx]
